# Habitat Shape Affects Polyploid Establishment in a Spatial, Stochastic Model

**DOI:** 10.3389/fpls.2020.592356

**Published:** 2020-11-16

**Authors:** Jonathan P. Spoelhof, Douglas E. Soltis, Pamela S. Soltis

**Affiliations:** ^1^Florida Museum of Natural History, University of Florida, Gainesville, FL, United States; ^2^Department of Biology, University of Florida, Gainesville, FL, United States

**Keywords:** dispersal, genome duplication, invasive species, polyploid establishment, spatial, stochastic model

## Abstract

Polyploidy contributes massively to the taxonomic and genomic diversity of angiosperms, but certain aspects of polyploid evolution are still enigmatic. The establishment of a new polyploid lineage following whole-genome duplication (WGD) is a critical step for all polyploid species, but this process is difficult to identify and observe in nature. Mathematical models offer an opportunity to study this process by varying parameters related to the populations, habitats, and organisms involved in the polyploid establishment process. While several models of polyploid establishment have been published previously, very few incorporate spatial factors, including spatial relationships between organisms, habitat shape, or population density. This study presents a stochastic, spatial model of polyploid establishment that shows how factors such as habitat shape and dispersal type can influence the fixation and persistence of nascent polyploids and modulate the effects of other factors. This model predicts that narrow, constrained habitats such as roadsides and coastlines may enhance polyploid establishment, particularly in combination with frequent clonal reproduction, limited dispersal, and high population density. The similarity between this scenario and the growth of many invasive or colonizing species along disturbed, narrow habitats such as roadsides may offer a partial explanation of the prevalence of polyploidy among invasive species.

## Introduction

Polyploid evolution is impossible to understand without knowing how and under what conditions new polyploid populations become established. Polyploid establishment is ephemeral and difficult to observe, so researchers have generally investigated this process through a combination of trait comparisons between diploids and recent polyploids, phylogenetic inference of trait evolution and ploidy, and mathematical models of the establishment process. Many traits associated with polyploidy (e.g., self-compatibility and perenniality) may be inherited from diploid progenitors, result from whole-genome duplication (WGD), or evolve after establishment (discussed below). Knowing the sequence of evolution in these traits is critical to understanding their potential contribution to establishment. Inferring that order is difficult, however, and consistent patterns may be clade-specific or dependent on other stochastic factors.

Polyploidy may facilitate the breakdown of self-incompatibility, particularly through genotype-specific effects (Mable, 2004). However, broad comparisons of diploid and polyploid taxa show minor or inconsistent associations between self-incompatibility, mating system and ploidy (Mable, 2004; Barringer, 2007), and phylogenetic studies of mating system and polyploidy in Solanaceae (Robertson et al., 2011; Zenil-Ferguson et al., 2019) have shown that self-compatibility is more likely to evolve prior to polyploidy than in concert with it. This trait is also frequently variable within species or even populations, which further complicates any inference performed at the species level. Polyploidy may also promote the expression of clonality (Van Drunen and Husband, 2018) or serve as a precursor for apomictic reproductive pathways (Hörandl and Hojsgaard, 2012; Hojsgaard et al., 2014; Hojsgaard and Hörandl, 2019). There is a strong association between polyploidy and clonality, yet phylogenetic analyses of evolutionary order (i.e., whether polyploidy precedes clonality or vice versa) have produced inconsistent results, particularly among clades (Herben et al., 2017; Van Drunen and Husband, 2019). Finally, polyploidy may spur the evolution of perenniality, possibly through a decrease in the rate of growth and development (as noted in Te Beest et al., 2012), but this is certainly not the rule. As with clonality, which is highly correlated with perenniality, inferring the order of evolution between these two traits yields inconclusive results (Van Drunen and Husband, 2019).

Mathematical models may provide the most general predictions about the factors and traits that facilitate polyploid establishment, at least in lieu of repeated, direct observations or tests of establishment itself. The model presented by Levin (1975) predicted that polyploid establishment is very unlikely for organisms that primarily outcross, while self-fertilization and clonal reproduction mitigate the mating disadvantage experienced by polyploids as the minority cytotype due to reproductive interference from diploids (minority cytotype exclusion, or MCE). This pattern recurs in other establishment models with variable parameters representing reproductive assurance, either through selfing (Rodriguez, 1996; Baack, 2005; Rausch and Morgan, 2005; Oswald and Nuismer, 2011; Fowler and Levin, 2016) or clonality (Chrtek et al., 2017), and the prevalence of at least some form of reproductive assurance across plant lineages with frequent recent and ancient polyploidy suggests that it is a critical driver of polyploid success (Spoelhof et al., 2019).

However, one very neglected area of polyploidy establishment modeling is spatial dynamics. Non-spatial models assume random spatial interactions between individuals in a population, which is almost necessarily violated in sessile organisms. Outcrossing, sessile organisms are more likely to mate with nearby individuals, and dispersal probability, while variable, will generally decrease proportionally with distance from that individual (Levin and Kerster, 1974). To our knowledge, one published model has focused on the interactions among polyploid and diploid plants in a fully spatial context (but see Li et al., 2004): Baack (2005) modeled the effects of selfing rate, polyploid advantage, and dispersal distance of pollen and seeds on the persistence of polyploids in a model parameterized on the species *Ranunculus adoneus*, which comprises diploid and autotetraploid cytotypes. One novel conclusion of this study was that the spatial effects of short seed dispersal distances increased the probability of polyploid persistence. Still, this model leaves many questions about the spatial aspects of polyploid establishment unanswered. How much do spatial effects contribute to polyploid establishment in a spatial model compared to a null (i.e., non-spatial) model? Do these effects interact with other parameters such as reproductive assurance or perenniality? How do habitat size and shape influence polyploid persistence?

This paper describes a new spatial, stochastic polyploid establishment model that addresses these questions. This model includes parameters for population size (*K*), reproductive assurance (*Ra*) through selfing or clonality, lifespan (annual vs. perennial), and habitat shape (square vs. narrow). The model simulates the introduction of a single autopolyploid to an otherwise diploid population, then tracks the length of time that cytotype polymorphism persists in the population and whether or not the polyploid cytotype is eventually fixed. Importantly, the model does not incorporate any trait or fitness differences between cytotypes (aside from the fact that they are reproductively isolated from each other, which can lead to reproductive interference when mating occurs between cytotypes), and each simulation is compared to a non-spatial control with the same starting conditions.

## Methods

### Model Construction

A stochastic, simulation-based model with parameters for habitat length and width (*Hl* and *Hw*, respectively), population size, individual lifespan (*Ls*), and reproductive assurance was developed in R (R Core Team, 2019). Two versions of the model were made to reflect different modes of reproductive assurance: a “clonal” model, and a “selfing” model. Both models begin by randomly populating a discrete spatial habitat of width *Hw* and length *Hl* with *K* individuals. For example, a habitat of *Hw* = *Hl* = 100 that contains *K* = 200 individuals would have a square shape, 10,000 discrete cells, and a population density of 2%. All individuals are self-compatible and able to act as either maternal (uniparentally or biparentally) or paternal parents of offspring. The spatial habitat is also occupied by a number of randomly placed, non-interactive individuals, such that the sum of *K* and the number of non-interactive individuals occupies 95% of the total cells in the habitat (this ratio was held constant in all simulations). Non-interactive individuals do not interact with simulated individuals (as described below) and are included to better represent a habitat that is co-occupied by other species and experiences a reasonable amount of disturbance (i.e., 5% of the habitat is available to new recruits in any given season). For example, if no non-interactive individuals were included, the model would represent a scenario where the habitat is completely open and none of the space has been occupied by other species. This is not realistic, even in frequently disturbed habitats. Individuals have staggered ages, so the habitat contains an equal number of individuals of each possible age (1 through *Ls*) during each simulated season. At the beginning of each season, all individuals of age *Ls* are removed from the population. Next, a maternal individual is randomly sampled from the population and produces a single offspring uniparentally with probability *Ra*. In the clonal model, this offspring can only disperse to an adjacent, empty position in the habitat. In the selfing model, the offspring disperses to an empty position in the habitat with a probability proportional to the inverse square of the distance between the maternal individual and the empty position. If uniparental reproduction does not occur (probability = 1 − *Ra*), a paternal individual is sampled from the rest of the population with a probability proportional to the inverse square of the distance between the maternal individual and the paternal individual. If the maternal and paternal individuals share the same cytotype, an outcrossed offspring is produced and dispersed to an empty position in the habitat with a probability proportional to the inverse square of the distance between the maternal individual and the empty position. If the maternal and paternal individuals do not share the same cytotype, they do not produce offspring. This process is repeated until the population reaches *K* individuals again, at which point the age of all individuals advances by one, the locations of the non-interactive individuals are randomized within the remaining cells, and the next season begins.

Initially, the simulated population is entirely diploid, and seasons proceed until the population turns over 50 times (i.e., 50 × *Ls* seasons). This period allows the initial diploid population to reach a spatial equilibrium. Visual plotting of the populations during this stage showed that the qualitative spatial distributions (e.g., highly clumped and evenly dispersed) that emerged from a given set of model parameters usually became stable before this point. Next, a single individual in the population is replaced with an autopolyploid, and the model proceeds until either diploids or polyploids reach fixation. Aside from their inability to mate with each other, diploids and polyploids are treated identically by the model. If fixation does not occur after the population turns over 1,000 times (1,000 × *Ls* seasons after the polyploid is introduced), cytotype polymorphism is considered stable and the simulation halts. For each simulation, the final ploidy of the population (diploid, polyploid, or polymorphic) is recorded along with the total number of seasons required to reach fixation, if it occurs.

For each parameter combination used in this study, a control simulation was performed using identical parameters and starting conditions. In control simulations, the probability of mating between any two individuals is not dependent on the distance between those individuals, and the probability of dispersal to any unoccupied cell is not dependent on the distance between the maternal individual and that cell. Therefore, control simulations are functionally non-spatial (even though they occur within the same spatial framework as the primary model) and simulate fully random mating between all individuals in a population. As most prior models of polyploid establishment assume random mating in a non-spatial framework, we included these simulations to identify the specific contributions of spatial factors to the predictions of this model.

### Simulation Conditions

A variety of parameter combinations was used to assess the model (Table 1). All possible combinations of these variables were simulated in the selfing model. In the clonal model, only the perennial lifespan (*Ls* = 10) was used because clonality is limited to perennial species. Two habitat shapes were used in this model: Square (*Hw*:*Hl* = 1:1) and narrow (*Hw*:*Hl* = 1:64). Square habitats were meant to represent relatively unconstrained, contiguous regions (e.g., a large forest tract), whereas narrow habitats were meant to represent highly constrained habitats such as road margins and coastlines. The spatial parameters were specified so that the effects of population density, habitat shape, and habitat size could be controlled (Table 1). For example, a population of 200 individuals in a narrow habitat (8 cells × 512 cells) could be compared to a population of 50 individuals in a habitat with the same shape and population density (4 cells × 256 cells).

**TABLE 1:** Establishment model parameters and values.

**Parameter**	**Description**	**Values**
*Hw*	Habitat width	Square: 32, 64 Narrow: 4, 8
*Hl*	Habitat length	Square: 32, 64 Narrow: 256, 512
*K*	Population carrying capacity	Small: 50 Large: 200
*Ls*	Lifespan (reproductive seasons + 1)	Annual: 2 Perennial: 10
*Ra*	Reproductive assurance (probability of uniparental reproduction)	Outcrossing: 0.05 Mostly outcrossing: 0.25 Mixed mating: 0.5 Mostly uniparental: 0.75 Uniparental: 0.95

We have included the code for this model in the Supplementary Material. Although R is not the most efficient environment for stochastic modeling, it is accessible to a wider academic audience in biology than other languages (e.g., C++). As a relatively simple model, this code could be used as an introductory resource for stochastic modeling, or to test hypotheses not considered in this paper. For example, any habitat that can be specified as a matrix with habitable and uninhabitable cells can be used, so users could test hypotheses about habitat fragmentation or specify habitats based on geographical locations of interest.

## Results

### Fixation Time

**FIGURE 1:**
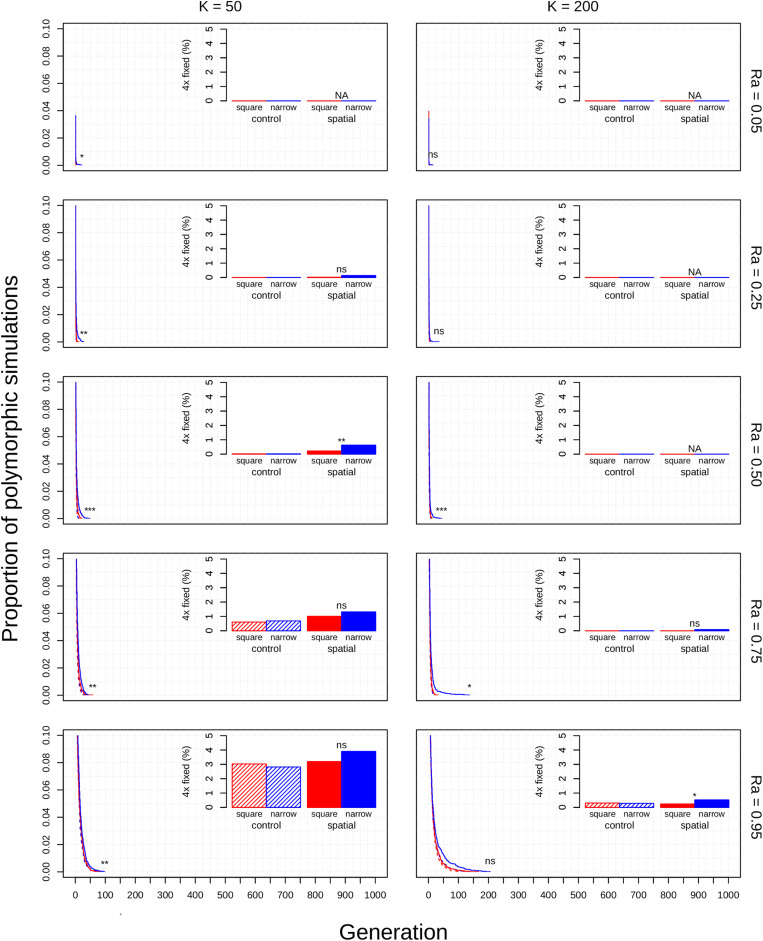
Polyploid persistence and fixation in simulated annual populations with selfing reproductive assurance. Values of *K* (columns) and *Ra* (rows) are noted in the margins. Each panel contains a survival plot showing the proportion of simulations that contained multiple cytotypes at each generation, and a bar plot showing the percentage of simulations that fixed as polyploid for each parameter combination. Results from non-spatial control simulations are shown as dashed lines and shaded bars, results from spatial simulations are shown as solid lines and solid bars, results from simulations in square habitats are shown in red, and results from simulations in narrow habitats are shown in blue. Asterisks denote the significance of log-rank tests (in survival plots) and Fisher tests (in bar plots) of differences between simulations in square and narrow habitats. Only results from simulations at a population density of 4.9% are shown. Significance: *ns* = *p* > 0.05; *p < 0.05; **p < 0.01; ***p < 0.001. See Supplementary Figure 1 in the Supplementary Material for a magnified view of the results from these simulations.

**FIGURE 2:**
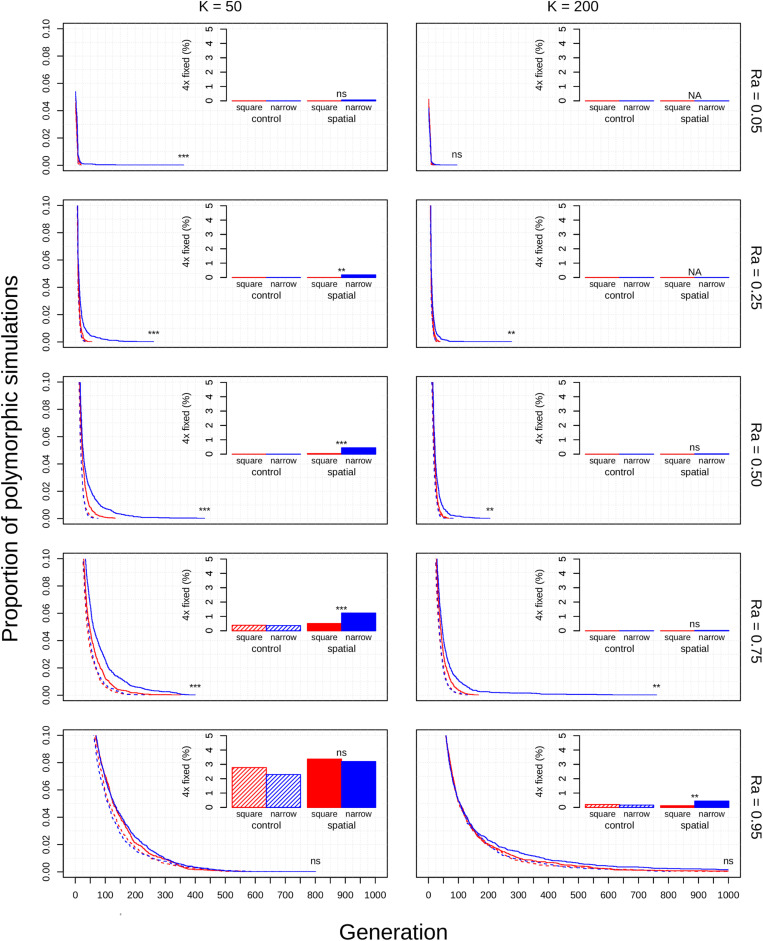
Polyploid persistence and fixation in simulated perennial populations with selfing reproductive assurance. Values of *K* (columns) and *Ra* (rows) are noted in the margins. Each panel contains a survival plot showing the proportion of simulations that contained multiple cytotypes at each generation, and a bar plot showing the percentage of simulations that fixed as polyploid for each parameter combination. Results from non-spatial control simulations are shown as dashed lines and shaded bars, results from spatial simulations are shown as solid lines and solid bars, results from simulations in square habitats are shown in red, and results from simulations in narrow habitats are shown in blue. Asterisks denote the significance of log-rank tests (in survival plots) and Fisher tests (in bar plots) of differences between simulations in square and narrow habitats. Only results from simulations at a population density of 4.9% are shown. Significance: *ns* = *p* > 0.05; *p < 0.05; **p < 0.01; ***p < 0.001.

**FIGURE 3:**
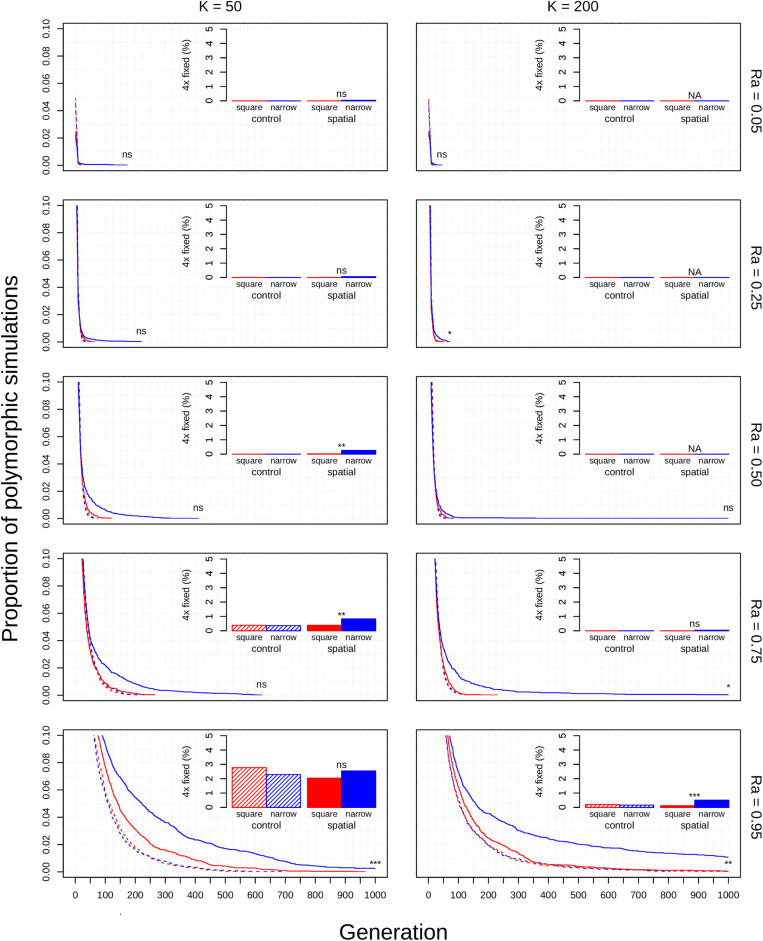
Polyploid persistence and fixation in simulated perennial populations with clonal reproductive assurance. Values of *K* (columns) and *Ra* (rows) are noted in the margins. Each panel contains a survival plot showing the proportion of simulations that contained multiple cytotypes at each generation, and a bar plot showing the percentage of simulations that fixed as polyploid for each parameter combination. Results from non-spatial control simulations are shown as dashed lines and shaded bars, results from spatial simulations are shown as solid lines and solid bars, results from simulations in square habitats are shown in red, and results from simulations in narrow habitats are shown in blue. Asterisks denote the significance of log-rank tests (in survival plots) and Fisher tests (in bar plots) of differences between simulations in square and narrow habitats. Only results from simulations at a population density of 4.9% are shown. Significance: *ns* = *p* > 0.05; *p < 0.05; ** *p* < 0.01; ***p < 0.001.

**FIGURE 4:**
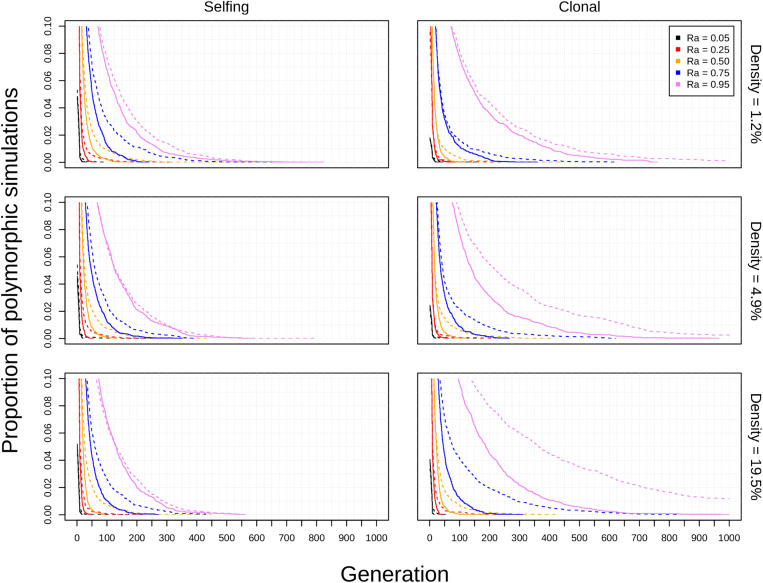
Effects of population density on polyploid persistence in spatial simulations (*K* = 50). Model type (columns) and population density (rows) are noted in the margins. Each panel contains a survival plot showing the proportion of simulations that contained multiple cytotypes at each generation, with varying values *Ra* represented by different colors (see legend). Results from simulations in square habitats are shown as solid lines, and results from simulations in narrow habitats are shown as dotted lines.

**FIGURE 5:**
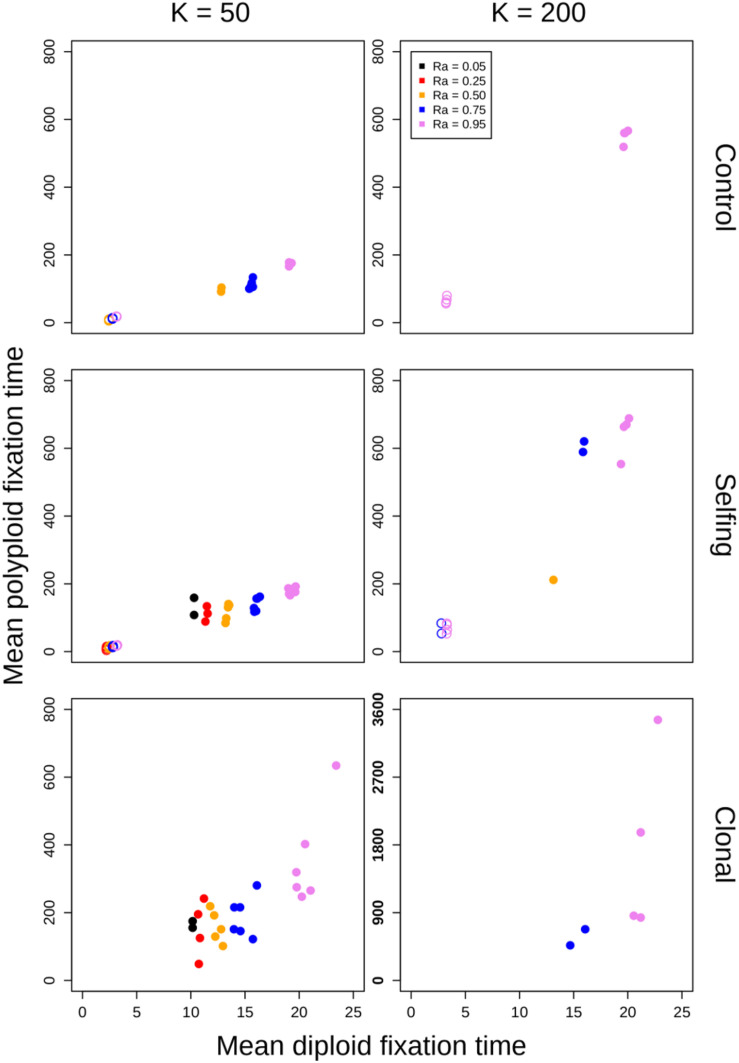
Polyploid fixation time vs. diploid fixation time. Values of *K* (columns) and model type (rows) are noted in the margins. In each panel, the geometric means of polyploid fixation time for all parameter combinations are plotted against the geometric means of diploid fixation time for all parameter combinations. Open circles represent simulations of annual populations, and closed circles represent simulations of perennial populations. Note the different *y*-axis scaling in the plot showing clonal simulations at *K* = 200.

The diploid cytotype becomes fixed very quickly in most simulations (Figures 1–3) because the initial proportion of polyploids is low [1/(*K* − 1)]. Higher reproductive assurance and perenniality consistently increased time to fixation (Figure 5). The effects of population size, population density, and habitat shape on time to fixation were more complex. Higher population size resulted in faster rates of fixation in earlier generations, but higher probabilities of polymorphism persistence in later generations (Figures 1–3), particularly in the clonal model and in simulations with higher reproductive assurance. Narrow habitat shape also generally increased time to fixation compared to square habitat shape and non-spatial controls (Figures 1–3). This effect appears to increase in proportion with population density in the clonal model, but diminishes at the highest level of reproductive assurance in the selfing model (Figure 4).

For simulations that resulted in polyploid fixation, time to fixation was still largely dependent on population size, reproductive assurance, and lifespan (Figure 5), but the results varied based on model type. The times to polyploid fixation were more variable in the selfing and clonal models than in the control model, and fixation times were much higher overall in simulations where *K* = 200 in the clonal model (Figure 5).

### Polyploid Fixation Probability

Polyploid fixation was highest in simulations with low population size and high reproductive assurance (Figures 1–3). In general, polyploid fixation was also higher in spatial simulations with narrow habitat shape. One exception to this trend is that spatial differences in polyploid fixation probability weaken or disappear with low population size and high reproductive assurance, particularly in perennial simulations of the selfing model (Figures 1–3).

## Discussion

### Caveats

The simulations used in this model do not include intercytotype gene flow or the formation of triploids from intercytotype crosses. These opportunities were not included primarily to simplify the model. The production of unreduced gametes and triploid fertility are both highly variable factors that are likely to be inconsistent across taxa and even throughout the timeline of establishment (Ramsey and Schemske, 1998; Köhler et al., 2010; Mason and Pires, 2015; Kreiner et al., 2017). This is also why each simulation included a single WGD, as the rate of spontaneous polyploid formation will depend on the same, highly variable factors. Both processes would act to favor the persistence and fixation of polyploids in the population, so measures of polyploid success are estimated conservatively in this study.

The inclusion of only a single WGD was also intentional for another reason. WGDs are rare events (Ramsey and Schemske, 1998), but established polyploid populations and species are typically the result of multiple formations (Soltis and Soltis, 1999). The long-term success of a polyploid lineage likely depends on multiple formations or gene flow from diploids (through unreduced gametes or odd-ploidy intermediates) as a means of developing genetic diversity within a population, which is vital for adaptation and response to selective pressures. By determining how long cytotype polymorphism persists in a population following a single WGD, these models can also estimate the relative likelihood that WGDs will overlap in the same population (or that rare, intercytotype gene flow will occur). In other words, conditions that favor longer polyploid persistence favor the accumulation of successive WGDs and genetic diversity among polyploids. While polyploid fixation is another measure of polyploid success in this study, rapid fixation will likely favor the production of genetically uniform polyploid populations that may be less adaptable in the long term.

Additionally, this model did not consider the effects of dispersal outside of the defined habitat. Offspring lost to dispersal outside of the suitable habitat would not affect the size of the population, as reproduction would still proceed until the population reached carrying capacity during each generation. Still, individuals along the periphery of their habitat would be less likely to achieve successful dispersal than those in the interior. The spatial difference between central and peripheral individuals is larger in square habitats, but any difference in this effect between square and narrow habitats would be dependent on average dispersal distance, which was not manipulated in this model. Further modeling could examine this effect by varying dispersal distance, as well as the geometric gradation between square and narrow habitats.

Finally, we did not include other important factors such as inbreeding depression, variable fitness or lifespan between cytotypes, prezygotic barriers between cytotypes, or differences in habitat disturbance. Our primary goal was to determine whether spatial dynamics (particularly habitat shape) could affect polyploid establishment in a simple, controlled framework. We acknowledge that these omissions limit the generality of our conclusions, and, as we noted in the methods, we encourage other researchers to build on this model framework and incorporate new factors or specify parameters based on their system of interest.

### Model Assessment

Each simulation of this model includes three critical phases. In the first phase, the initial polyploid must reproduce at least once during its lifespan. If that happens, the simulation reaches the second phase, where there are multiple polyploids in the population that can reproduce uniparentally or biparentally. If polyploids are able to reproduce until they reach half of the population carrying capacity, the population reaches the third phase: a tipping point where there is an equal chance that polyploids and diploids will reach fixation because they are treated neutrally by the model. The first and second phases are critical in determining the likelihood of polyploid fixation. Factors that act to reduce MCE during these phases increase the probability that polyploids will reach parity with diploids in the population.

The non-spatial parameters of this model all had substantial impacts on the rate of polyploid fixation or the time to fixation. High reproductive assurance directly reduces polyploid MCE by decreasing the probability of reproductive interference (Figures 1–3). Reproductive assurance is especially critical during the first phase of each simulation; without the possibility of intercytotype crossing, the first polyploid must rely on uniparental reproduction to establish a breeding population. Simulations with higher reproductive assurance allow polyploids to reproduce more quickly in phase two and lose fewer reproductive opportunities to intercytotype crosses. These effects are equally present in the third phase, where they reduce the chance that either cytotype will go extinct by reducing the proportional effects of MCE. This stabilizing effect is reflected in the longer times to fixation observed in simulations with high reproductive assurance (Figure 5). Alternatively, low reproductive assurance caused nearly all populations to fix as diploid within a short period of time due to the unmitigated effects of MCE (Figures 1–3). Low population size also directly reduces polyploid MCE, but through a different mechanism: by increasing the initial proportion of polyploids in the population. In small populations, less reproduction is required for the initial polyploid to reach parity with diploids and accelerates the process of cytotype drift in phase three. Rates of polyploid fixation are therefore much higher in simulations with low population sizes (Figures 1–3). While annual lifespan did not greatly impact the rate of polyploid fixation, it did greatly decrease the time to fixation (Figure 5). This makes intuitive sense: annual populations turn over much more quickly, so the minimum time required for either diploids or polyploids to reach fixation is reduced. This distinction may be important in explaining the differential frequency of occurrence of polyploids among annuals and perennials in nature—polyploidy appears to be much more common in herbaceous perennials than in annuals (Stebbins, 1938; Husband et al., 2013; Van Drunen and Husband, 2019). If rapid fixation in annuals reduces the probability of overlapping WGDs and intercytotype gene flow within the population, then annual polyploid populations may be more prone to extinction because of a lack of genetic diversity and eventual deterioration through inbreeding depression or the accumulation of deleterious alleles.

The theoretical impacts of reproductive assurance and population size on polyploid establishment have been examined in previously published deterministic and stochastic models (Rodriguez, 1996; Baack, 2005; Rausch and Morgan, 2005; Oswald and Nuismer, 2011; Fowler and Levin, 2016; Chrtek et al., 2017), and the results presented here are qualitatively similar to the results of those studies. However, these factors have not been studied extensively within a spatial context (aside from Baack, 2005). The selfing and clonal models generally relaxed constraints on polyploid fixation, particularly regarding reproductive assurance. In the control model, no polyploid fixation was seen at *Ra* < 0.5 in populations of 50 individuals and at *Ra* < 0.95 in populations of 200 individuals. In the selfing and clonal models, polyploid fixation was observed at all values of *Ra* in populations of 50 individuals and at *Ra* > 0.5 in populations of 200 individuals. This effect is likely due to the spatial dynamics of mating and dispersal—assortative mating among cytotypes is more likely when dispersal is limited (Li et al., 2004; Baack, 2005), which mitigates the effects of MCE that polyploids experience in the initial phases of establishment. However, this effect varied with habitat shape in both spatial models. Most of the increase in polyploid fixation in spatial models came from simulations in narrow habitats; square habitats often had rates of polyploid fixation similar to or slightly larger than non-spatial control simulations (Figures 1–3). This effect likely results from an amplification of assortative mating in habitats that are more spatially restricted, especially if dispersal is limited, too. Narrow habitats also generally increased time to fixation, with particularly pronounced effects in the clonal model (Figure 3). Because the offspring of simulated clonal organisms could only disperse to adjacent cells, each cytotype could effectively block an entire portion of the narrow habitat. This spatial organization stabilizes polymorphism within the population, and the effect increased with greater population density within the clonal model (Figure 4).

### Significance

Spatial effects may relax constraints on polyploid establishment by causing non-random mating and dispersal. In the selfing and clonal models described above, factors that limited the spatial probabilities of mating and dispersal generally counteracted the effects of MCE. These factors included a clonal mode of reproductive assurance and narrow habitat shape, in particular.

Both clonal reproduction and selfing directly counteract MCE, but clonal reproduction results in more closely spaced groups of plants. If these plants are also capable of outcrossing, they are much more likely to mate with their own clonal offspring than with other individuals in the population. In the context of a polycytotypic population, this results in assortative mating among diploids and polyploids, respectively. It also decreases the probability that the offspring of another cytotype will disperse into a “clump” of plants and begin to displace them. Polyploidy and clonality are very strongly associated in angiosperms (Herben et al., 2017; Van Drunen and Husband, 2019). While there is a possibility that WGD may induce or enhance clonality (Van Drunen and Husband, 2019), this study demonstrates that preexisting clonal traits may facilitate polyploid establishment, even when these traits do not differ between diploids and polyploids. Unlike selfing, clonality could allow polyploids to persist in a population without experiencing the effects of inbreeding depression (which was not considered in this study), possibly increasing the relative chance that they will avoid extinction long enough to overlap with subsequent WGDs in the population.

The effects of habitat shape have not been examined in previous models of polyploid establishment, but it varies widely in both natural and disturbed areas. Coastlines, riverbanks, cliff sides, roadsides, railways, and agricultural margins are all examples of relatively linear habitats whose communities tend to differ from those in surrounding areas. In these cases, suitable habitat may be restricted in one dimension, so only dispersal along a narrow corridor will be successful. The results of this study suggest that narrow habitats can increase the probability of polyploid fixation and delay the fixation of any cytotype following WGD, and that these effects may be particularly pronounced in highly clonal, high-density populations. Populations of many invasive angiosperm species often share these characteristics, and they are also more likely to be polyploid (Pandit et al., 2011; Te Beest et al., 2012). The association between polyploidy and invasiveness has usually been supported with adaptive explanations, including that polyploids will have broader ecological niches, different reproductive traits, or increased vigor when compared to their diploid progenitors (Prentis et al., 2008; Te Beest et al., 2012). While these explanations certainly hold true in some cases, there may be an additional, neutral explanation for the enrichment of polyploidy among invasive species. The disturbance associated with roads and railways provides invasive species with exploitable habitat, and the linear, interconnected nature of these habitats also allows these species to spread to new areas. Both of these factors are critical in the establishment of many invasive species (e.g., Mortensen et al., 2009; Joly et al., 2011; Dar et al., 2015). We have shown that long, narrow habitats may enhance polyploid establishment and fixation without invoking any difference between the characteristics of diploid and polyploid cytotypes. If this model is representative of nature, one would expect invasive plants that exploit narrow habitats to be (1) more frequently polyploid or polycytotypic, and (2) more frequently polyploid in their invaded habitat than in their natural habitat (assuming their natural habitat is not often “narrow”). Both of these predictions are supported by observational studies in several species (Pyšek et al., 2009; Pandit et al., 2011; Te Beest et al., 2012). Furthermore, *Tragopogon miscellus* and *Senecio cambrensis*, some of the best-studied examples of recently formed polyploid species, arose through hybridization of introduced species and were discovered along disturbed, often narrow habitats such as railway margins and roadsides (Ownbey, 1950; Novak et al., 1991; Abbott and Lowe, 2004). Another well-studied and recently formed polyploid, *Spartina anglica*, also arose through hybridization of an introduced species and spread along a different type of narrow habitat—coastlines in the United Kingdom (Gray et al., 1991). Notably, each of these species is capable of reproductive assurance (via self-fertilization in *T. miscellus* and *S. cambrensis* and extensive clonal spread in *S. anglica*), which, according to this model and others, can also facilitate polyploid establishment. Therefore, disentangling the contributions of habitat shape, reproductive traits, and disturbance to polyploid establishment is difficult. One way to isolate these factors would be to intensively measure ploidy variation (e.g., via flow cytometry) in plant communities occupying natural, linear habitats and comparing the results to nearby, less-linear communities. For example, the effects of habitat shape observed in our model would be indirectly corroborated if (1) polyploidy is more common in a riverside community than in an adjacent forest community, and (2) both communities experience minimal human disturbance and have similar distributions of reproductive traits. In general, increased efforts to identify ploidy variation and recently formed polyploids in nature will be essential for testing the predictions of this study and other polyploid establishment models.

## Data Availability Statement

The original contributions presented in the study are included in the article/Supplementary Material; further inquiries can be directed to the corresponding author.

## Author Contributions

JS developed the model, analyzed model output, and prepared the manuscript. DS and PS provided critical revisions to the manuscript. All authors contributed to the article and approved the submitted version.

## Conflict of Interest

The authors declare that the research was conducted in the absence of any commercial or financial relationships that could be construed as a potential conflict of interest.
