# Supplementary material for: Habitat Shape Affects Polyploid Establishment in a Spatial, Stochastic Model
Source: Front Plant Sci. 2020 Nov 16;11:592356. doi: 10.3389/fpls.2020.592356 (PMC7701104; doi:10.3389/fpls.2020.592356)
Supplement: Supplementary file 1 [file Data_Sheet_1.DOCX]

Supplementary Material

# Supplementary Figures


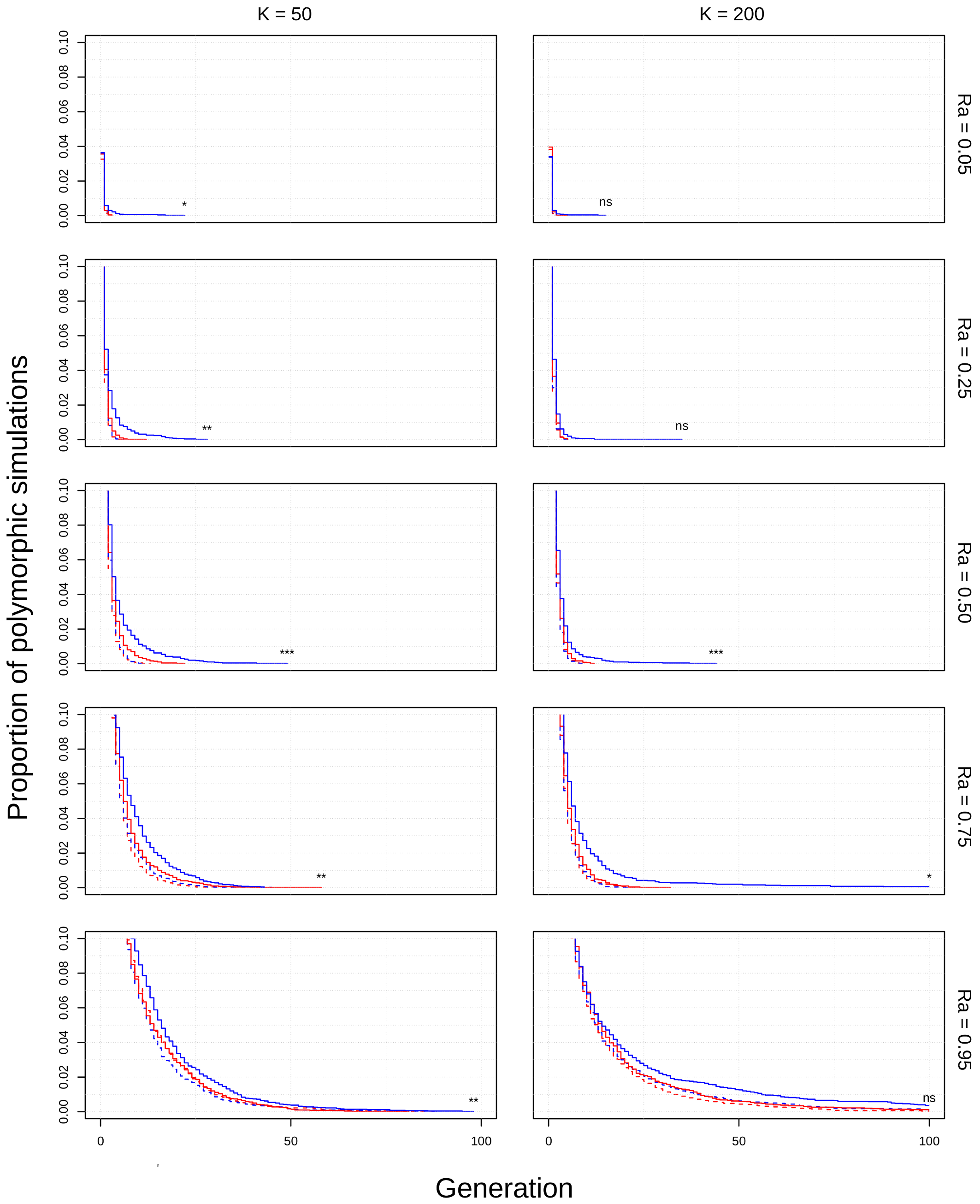


**Figure S1.** Polyploid persistence and fixation in simulated annual populations with selfing reproductive assurance. Values of K (columns) and Ra (rows) are noted in the margins. Each panel contains a survival plot showing the proportion of simulations that contained multiple cytotypes at each generation. Results from nonspatial control simulations are shown as dashed lines, results from spatial simulations are shown solid lines, results from simulations in square habitats are shown in red, and results from simulations in narrow habitats are shown in blue. Asterisks denote the significance of log-rank tests of differences between simulations in square and narrow habitats. Only results from simulations at a population density of 4.9% are shown. Significance: ns = p > 0.05; * = p < 0.05; ** = p < 0.01; *** p < 0.001.

# Model Code

#The following R code defines three functions that correspond to the control (function ‘control_model’), selfing (function ‘selfing_model’), and clonal (function ‘clonal_model’) models that are described in the text. Example function calls are included at the end of the document.

#Load package ‘Rcpp’

library(Rcpp)

#####generate function ('probr') to calculate distance-based probability vectors for mating, dispersal (requires package ‘Rcpp’)#####

cppFunction("

NumericVector probr(NumericVector dis) {

NumericVector bob = 1/pow(dis,2);

NumericVector al = bob/sum(bob);

return al;

}")

#####Control model - Spatially random mating and dispersal#####

#function inputs

control_model = function(K, #population size (must be a multiple of Ls)

Ls, #organism lifespan (equals reproductive seasons + 1)

Hl, #habitat length

Hw, #habitat width

Ra, #probability of uniparental reproduction (must be nonzero)

sims = 100, #number of simulations

seed = sample(1:1000000,1) #seed for simulations

){

#generate vector to record generations to cytotype fixation after the polyploid is introduced

gens = vector("numeric", sims)

#generate list to record which cytotype was fixed

ploids = as.list(rep(NA, sims))

#generate habitat matrix based on Hl and Hw

habitat = matrix(NA, nrow = Hl, ncol = Hw)

#generate a vector of all possible habitat positions

hab_pos = 1:sum(is.na(habitat))

#specify deaths per generation (equals population turnover divided by lifespan)

death_count = K/Ls

#specify how many times the diploid population should turn over during burn-in

burnin = 50*Ls

#set simulation seed

set.seed(seed)

#generate n = sims lists of population starting positions

pops = replicate(sims, cbind(ind_ploid = 2, ind_pos = sample(hab_pos, K, replace = F)), simplify = F)

#generate empty vector template to contain positions of dead individuals in any given generation

new_pos_empty = rep(NA, death_count)

#begin simulation

for(i in 1:sims){

#select population based on i

pop = pops[[i]]

#repeat death/reproduction cycle with only diploids for n = burnin generations

for(j in 1:burnin){

#n = death_count individuals die at the beginning of the generation

pop = pop[-(1:death_count),]

#n = death_count individuals are randomly selected to replace the dead individuals

lucky_pos = sample(pop[,2], death_count, replace = T)

#specify all empty positions

emptys = hab_pos[-pop[,2]]

#limit empty space to 5% of total population to simulate a habitat coocupied by other species

emptys_lim = sample(emptys, as.integer(K + (0.05*length(hab_pos))), replace = F)

#generate empty vector for offspring positions

new_pos = new_pos_empty

#generate empty vector to record filled positions

picks = new_pos_empty

#loop to replace dead individuals

for(k in 1:length(new_pos)){

if(k==1){

#positions of new individuals selected at random

pick = sample(1:length(emptys_lim), 1, replace = T)

}else{

skip = picks[!is.na(picks)]

pick = sample((1:length(emptys_lim))[-skip], 1, replace = T)

}

new_pos[k] = emptys_lim[pick]

picks[k] = pick

}

#replace dead positions with new offspring positions

pop = rbind(pop, cbind(2,new_pos))

}

#convert a single individual from diploid to polyploid

pop[K,1] = 4

#set generation to zero

gen = 0

#proceed until until one cytotype reaches fixation or the population turns over 1000 times

while(length(unique(pop[,1])) == 2 && gen < ((1000*Ls)+Ls)){

#advance generation

gen = gen + 1

#n = death_count individuals die at the beginning of the generation

pop = pop[-(1:death_count),]

#limit empty space to 5% of total population to simulate a habitat coocupied by other species

hab_pos_lim = sample(hab_pos[-pop[,2]], as.integer(K + (0.05*length(hab_pos))), replace = F)

#reproduction restores population size to K

while(nrow(pop) < K){

#randomly select an individual to mate

lucky = sample(1:nrow(pop), 1, replace = T)

#find its ploidy...

ploid = pop[lucky,1]

#...and its position

lucky_pos = pop[lucky,2]

#sample to determine whether uniparental reproduction occurs

TF_Ra = sample(c(T,F), 1, prob = c(Ra, 1-Ra), replace = T)

#if a spatially sampled mate is the same ploidy as lucky OR lucky reproduces uniparentally, create a new individual

if(TF_Ra == F && sample(pop[-lucky,1], 1, replace = T) == ploid){

#specify empty positions

emptys_lim = hab_pos_lim[!(hab_pos_lim %in% pop[,2])]

#randomly select an empty position for dispersal

new_pos = sample(emptys_lim, 1, replace = T)

#add new individual to habitat and population dataframe

pop = rbind(pop,c(ploid,new_pos))

}else if(TF_Ra == T){

#specify empty positions

emptys = hab_pos_lim[!(hab_pos_lim %in% pop[,2])]

#randomly select an empty position for dispersal

new_pos = sample(emptys_lim, 1, replace = T)

#add new individual to habitat and pop dataframe

pop = rbind(pop,c(ploid,new_pos))

}

}

}

#output run data

gens[i] = gen

ploids[[i]] = unique(pop[,1])

print(i)

}

#compile results in matrix

res = cbind(gens, ploids = unlist(lapply(ploids, sum)))

#function output

return(res)

}

#####Selfing model - mating and dispersal probability follow inverse-square spatial relationship#####

#function inputs

selfing_model = function(K, #population size (must be a multiple of Ls)

Ls, #organismal lifespan (equals reproductive seasons + 1)

Hl, #habitat length

Hw, #habitat width

Ra, #probability of uniparental reproduction (must be nonzero)

sims = 100, #number of simulations

seed = sample(1:1000000,1) #seed for simulations

){

#generate vector to record generations to cytotype fixation after the polyploid is introduced

gens = vector("numeric", sims)

#generate list to record which cytotype was fixed

ploids = as.list(rep(NA, sims))

#generate habitat matrix based on Hl and Hw

habitat = matrix(NA, nrow = Hl, ncol = Hw)

#generate a vector of all possible habitat positions

hab_pos = 1:sum(is.na(habitat))

#get position coordinates

pos = which(is.na(habitat), arr.ind = T)

#generate matrix of distances between all possible positions

d = as.matrix(dist(pos, diag = T, upper = T))

#convert distances to integers for efficiency

d_int = matrix(as.integer(round(d*10,0)), nrow = nrow(d), ncol = ncol(d))

#specify deaths per generation (equals population turnover divided by lifespan)

death_count = K/Ls

#specify how many times the diploid population should turn over during burn-in

burnin = 50*Ls

#set simulation seed

set.seed(seed)

#generate n = sims lists of population starting positions

pops = replicate(sims, cbind(ind_ploid = 2, ind_pos = sample(hab_pos, K, replace = F)), simplify = F)

#generate empty vector template to contain positions of dead individuals in any given generation

new_pos_empty = rep(NA, death_count)

#begin simulation

for(i in 1:sims){

#select population based on i

pop = pops[[i]]

#repeat death/reproduction cycle with only diploids for n = burnin generations

for(j in 1:burnin){

#n = death_count individuals die at the beginning of the generation

pop = pop[-(1:death_count),]

#n = death_count individuals are randomly selected to replace the dead individuals

lucky_pos = sample(pop[,2], death_count, replace = T)

#specify all empty positions

emptys = hab_pos[-pop[,2]]

#limit empty space to 5% of total population to simulate a habitat coocupied by other species

emptys_lim = sample(emptys, as.integer(K + (0.05*length(hab_pos))), replace = F)

#sample matrix of distances from positions in lucky_pos to empty spaces

empty_dists = as.matrix(d_int[emptys_lim,lucky_pos])

#generate empty vector for offspring positions

new_pos = new_pos_empty

#generate empty vector to record filled positions

picks = new_pos_empty

#loop to replace dead individuals

for(k in 1:length(new_pos)){

if(k==1){

#positions of new individuals selected probabilistically based on distance from positions in lucky_pos

pick = sample(1:length(emptys_lim), 1, prob = probr(empty_dists[,k]), replace = T)

}else{

skip = picks[!is.na(picks)]

pick = sample((1:length(emptys_lim))[-skip], 1, prob = probr(empty_dists[,k][-skip]), replace = T)

}

new_pos[k] = emptys_lim[pick]

picks[k] = pick

}

#replace dead positions with new offspring positions

pop = rbind(pop, cbind(2,new_pos))

}

#convert a single individual from diploid to polyploid

pop[K,1] = 4

#set generation to zero

gen = 0

#proceed until until one cytotype reaches fixation or the population turns over 1000 times

while(length(unique(pop[,1])) == 2 && gen < ((1000*Ls)+Ls)){

#advance generation

gen = gen + 1

#n = death_count individuals die at the beginning of the generation

pop = pop[-(1:death_count),]

#limit empty space to 5% of total population to simulate a habitat coocupied by other species

hab_pos_lim = sample(hab_pos[-pop[,2]], as.integer(K + (0.05*length(hab_pos))), replace = F)

#reproduction restores population size to K

while(nrow(pop) < K){

#randomly select an individual to mate

lucky = sample(1:nrow(pop), 1, replace = T)

#find its ploidy...

ploid = pop[lucky,1]

#...and its position

lucky_pos = pop[lucky,2]

#find distances from lucky to all potential mates

luckys_mates = d_int[pop[-lucky,2],lucky_pos]

#sample to determine whether uniparental reproduction occurs

TF_Ra = sample(c(T,F), 1, prob = c(Ra, 1-Ra), replace = T)

#if a spatially sampled mate is the same ploidy as lucky OR lucky reproduces uniparentally, create a new individual

if(TF_Ra == F && sample(pop[-lucky,1], 1, prob = probr(luckys_mates), replace = T) == ploid){

#specify empty positions

emptys = hab_pos_lim[!(hab_pos_lim %in% pop[,2])]

#find distances from lucky to all empty positions

empty_dists = d_int[emptys, lucky_pos]

#select an empty position for dispersal based on distances in empty_dists

new_pos = sample(emptys, 1, prob = probr(empty_dists), replace = T)

#add new individual to habitat and population dataframe

pop = rbind(pop,c(ploid,new_pos))

}else if(TF_Ra == T){

#specify empty positions

emptys = hab_pos_lim[!(hab_pos_lim %in% pop[,2])]

#find distances from lucky to all empty positions

empty_dists = d_int[emptys, lucky_pos]

#select an empty position for dispersal based on distances in empty_dists

new_pos = sample(emptys, 1, prob = probr(empty_dists), replace = T)

#add new individual to habitat and population dataframe

pop = rbind(pop,c(ploid,new_pos))

}

}

}

#output run data

gens[i] = gen

ploids[[i]] = unique(pop[,1])

print(i)

}

#compile results in matrix

res = cbind(gens, ploids = unlist(lapply(ploids, sum)))

#function output

return(res)

}

#####Clonal model - uniparental dispersal limited to adjacent cells#####

#function inputs

clonal_model = function(K, #population size (must be a multiple of Ls)

Ls, #organismal lifespan (equals reproductive seasons + 1)

Hl, #habitat length

Hw, #habitat width

Ra, #probability of uniparental reproduction (must be nonzero)

sims = 100, #number of simulations

seed = sample(1:1000000,1) #seed for simulations

){

#generate vector to record generations to cytotype fixation after the polyploid is introduced

gens = vector("numeric", sims)

#generate list to record which cytotype was fixed

ploids = as.list(rep(NA, sims))

#generate habitat matrix based on Hl and Hw

habitat = matrix(NA, nrow = Hl, ncol = Hw)

#generate a vector of all possible habitat positions

hab_pos = 1:sum(is.na(habitat))

#get position coordinates

pos = which(is.na(habitat), arr.ind = T)

#generate matrix of distances between all possible positions

d = as.matrix(dist(pos, diag = T, upper = T))

#convert distances to integers for efficiency

d_int = matrix(as.integer(round(d*10,0)), nrow = nrow(d), ncol = ncol(d))

#specify deaths per generation (equals population turnover divided by lifespan)

death_count = K/Ls

#specify how many times the diploid population should turn over during burn-in

burnin = 50*Ls

#set simulation seed

set.seed(seed)

#generate n = sims lists of population starting positions

pops = replicate(sims, cbind(ind_ploid = 2, ind_pos = sample(hab_pos, K, replace = F)), simplify = F)

#begin simulation

for(i in 1:sims){

#select population based on i

pop = pops[[i]]

#repeat death/reproduction cycle with only diploids for n = burnin generations

for(j in 1:burnin){

#n = death_count individuals die at the beginning of the generation

pop = pop[-(1:death_count),]

#limit empty space to 5% of total population to simulate a habitat coocupied by other species

hab_pos_lim = sample(hab_pos[-pop[,2]], as.integer(K + (0.05*length(hab_pos))), replace = F)

#reproduction restores population size to K

while(nrow(pop) < K){

#randomly select an individual to mate

lucky = sample(1:nrow(pop), 1, replace = T)

#find its ploidy...

ploid = pop[lucky,1]

#...and its position

lucky_pos = pop[lucky,2]

#sample to determine whether clonal reproduction occurs

TF_Ra = sample(c(T,F), 1, prob = c(Ra, 1-Ra), replace = T)

#If a (probably nearby) chosen mate is the same ploidy OR lucky expresses Ra, create a new individual

if(TF_Ra == F){

#specify empty positions

emptys = hab_pos_lim[!(hab_pos_lim %in% pop[,2])]

#find distances from lucky to all empty positions

empty_dists = d_int[emptys, lucky_pos]

#select an empty position for dispersal based on distances in empty_dists

new_pos = sample(emptys, 1, prob = probr(empty_dists), replace = T)

#add new individual to habitat and population dataframe

pop = rbind(pop,c(ploid,new_pos))

}else if(TF_Ra == T){

#specify empty positions

emptys = hab_pos_lim[!(hab_pos_lim %in% pop[,2])]

#find distances from lucky to all empty positions

empty_dists = d_int[emptys, lucky_pos]

#find all empty positions that are adjacent to lucky

adjacent = which(empty_dists <= as.integer(round(sqrt(2)*10,0)) & empty_dists != 0)

#if more than one adjacent position is empty...

if(length(adjacent)>1){

#...select one of those empty positions at random for clonal dispersal

new_pos = sample(emptys[adjacent], 1, prob = probr(empty_dists[adjacent]), replace = T)

#add new individual to habitat and population dataframe

pop = rbind(pop,c(ploid,new_pos))

#if only one adjacent position is empty...

}else if(length(adjacent) == 1){

#...select that position for clonal dispersal

new_pos = emptys[adjacent]

#add new individual to habitat and population dataframe

pop = rbind(pop,c(ploid,new_pos))

}

}

}

}

#convert a single individual from diploid to polyploid

pop[K,1] = 4

#set generation to zero

gen = 0

#proceed until until one cytotype reaches fixation or the population turns over 1000 times

while(length(unique(pop[,1])) == 2 & gen < ((1000*Ls)+Ls)){

#advance generation

gen = gen + 1

#n = death_count individuals die at the beginning of the generation

pop = pop[-(1:death_count),]

#limit empty space to 5% of total population to simulate a habitat coocupied by other species

hab_pos_lim = sample(hab_pos[-pop[,2]], as.integer(K + (0.05*length(hab_pos))), replace = F)

#reproduction restores population size to K

while(nrow(pop) < K){

#randomly select an individual to mate

lucky = sample(1:nrow(pop), 1, replace = T)

#find its ploidy...

ploid = pop[lucky,1]

#...and its position

lucky_pos = pop[lucky,2]

#find distances from lucky to all potential mates

luckys_mates = d_int[pop[-lucky,2],lucky_pos]

#sample to determine whether uniparental reproduction occurs

TF_Ra = sample(c(T,F), 1, prob = c(Ra, 1-Ra), replace = T)

#if a spatially sampled mate is the same ploidy as lucky OR lucky reproduces uniparentally, create a new individual

if(TF_Ra == F && sample(pop[-lucky,1], 1, prob = probr(luckys_mates), replace = T) == ploid){

#specify empty positions

emptys = hab_pos_lim[!(hab_pos_lim %in% pop[,2])]

#find distances from lucky to all empty positions

empty_dists = d_int[emptys, lucky_pos]

#select an empty position for dispersal based on distances in empty_dists

new_pos = sample(emptys, 1, prob = probr(empty_dists), replace = T)

#add organism to habitat and population dataframe

pop = rbind(pop,c(ploid,new_pos))

}else if(TF_Ra == T){

#specify empty positions

emptys = hab_pos_lim[!(hab_pos_lim %in% pop[,2])]

#find distances from lucky to all empty positions

empty_dists = d_int[emptys, lucky_pos]

#find all empty positions that are adjacent to lucky

adjacent = which(empty_dists <= as.integer(round(sqrt(2)*10,0)) & empty_dists != 0)

#if more than one adjacent position is empty...

if(length(adjacent) > 1){

#...select one of those empty positions at random for clonal dispersal

new_pos = sample(emptys[adjacent], 1, prob = probr(empty_dists[adjacent]), replace = T)

#add new individual to habitat and population dataframe

pop = rbind(pop,c(ploid,new_pos))

#if only one adjacent position is empty...

}else if(length(adjacent) == 1){

#...select that position for clonal dispersal

new_pos = emptys[adjacent]

#add new individual to habitat and population dataframe

pop = rbind(pop,c(ploid,new_pos))

}

}

}

}

#output run data

gens[i] = gen

ploids[[i]] = unique(pop[,1])

print(i)

}

#compile results in matrix

res = cbind(gens, ploids = unlist(lapply(ploids, sum)))

#function output

return(res)

}

#####running the model#####

#specify population size

K = 10

#specify lifespan (must be a factor of population size)

Ls = 2

#specify probability of uniparental reproduction

Ra = 0.5

#Specify habitat length and width

Hl = 10

Hw = 10

#specify number of simulations (defaults to 100 simulations)

sims = 100

#specify starting seed (optional)

seed = 12345

#run each model with the same parameters and starting conditions

control_model(K = K,

Ls = Ls,

Ra = Ra,

Hl = Hl,

Hw = Hw,

sims = sims,

seed = seed)

selfing_model(K = K,

Ls = Ls,

Ra = Ra,

Hl = Hl,

Hw = Hw,

sims = sims,

seed = seed)

clonal_model(K = K,

Ls = Ls,

Ra = Ra,

Hl = Hl,

Hw = Hw,

sims = sims,

seed = seed)

#output is a table with nrow = sims and columns specifying:

# 1) the total elapsed generations between polyploid formation and cytotype fixation (min = Ls, max = 10,000 generations)

# 2) which ploidy was fixed. 2 = diploid, 4 = tetraploid, 6 = mixed (i.e. if fixation does not occur before 10,000 generations)
